# Effects of long-term proBNP cardiac gene delivery in experimental hypertensive heart disease

**DOI:** 10.1186/1471-2210-11-S1-O16

**Published:** 2011-08-01

**Authors:** Alessandro Cataliotti, Jason M Tonne, Guido Boerrigter, Salvatore Mangiafico, John C Burnett, Yasuhiro Ikeda

**Affiliations:** 1 Mayo Clinic, Cardiorenal Research Laboratory, 200 First St SW, Rochester, MN 55905, USA

## Background

In the current study, we tested the effects of sustained cardiac proBNP gene delivery on blood pressure (BP), cardiac function and remodeling in spontaneously hypertensive rats (SHR).

## Methods

We used the myocardium-tropic adeno-associated virus serotype 9 (AAV9) vector to achieve continuously enhanced cardiac rat proBNP expression. *p<0.05 vs treated rats.

## Results

In SHR, a single systemic administration of AAV9 vector allowed longterm, cardiac BNP overexpression with an increase in plasma BNP levels as compared with untreated SHR (433.3±17 vs 60.6±15* pg/ml). This further resulted in reductions in systolic (121±11 vs 184±13* mm Hg) and diastolic (103±5 vs 148±24* mm Hg) BP for nine months after injection as compared with untreated SHR. Left ventricular (LV) thickness (1.87±0.1 vs 2.16±0.3* mm), LV end-systolic dimensions (3.96±0.3 vs 4.66±0.6* mm) and LV mass (0.4±0.01 vs 0.49±0.01* gr) were reduced, while ejection fraction was increased (83±2 vs 74±4* %) in BNP-treated compared to untreated SHR. Circumferential systolic strain (-5.04±0.4 vs -3.74±0.4* %) and strain rate of the early phase of diastole (-4.57±2.1 vs -2.41±1.3 1/s) were improved in BNP-treated compared with controls. Importantly, the improvement in cardiac function and structure also resulted in a significant increase in survival (p<0.001 vs untreated SHR). Non-cardiac overexpression of BNP, via AAV2 vector, was not associated with changes in plasma BNP and BP in SHR. Nevertheless, normotensive Wistar rats injected with AAV9 proBNP vector showed significantly reduced heart/body weights (0.31± 0.1 vs 3.9±0.1* %) four weeks after injection without BP reduction compared to untreated rats.

## Conclusion

AAV9 vector facilitates sustained cardiac proBNP overexpression improves both systolic and diastolic cardiac function in hypertensive heart disease. Long-term proBNP delivery improved survival in SHR. The effects on cardiac structure and function occurred independently of BP lowering effects in normotensive Wistar rats.

